# Ontogeny of cardiomyocytes: ultrastructure optimization to meet the demand for tight communication in excitation–contraction coupling and energy transfer

**DOI:** 10.1098/rstb.2021.0321

**Published:** 2022-11-21

**Authors:** Rikke Birkedal, Martin Laasmaa, Jelena Branovets, Marko Vendelin

**Affiliations:** Laboratory of Systems Biology, Department of Cybernetics, Tallinn University of Technology, Akadeemia 15, room SCI-218, 12618 Tallinn, Estonia

**Keywords:** cardiomyocytes, energy transfer, excitation–contraction coupling, heart, intracellular diffusion, ontogeny

## Abstract

The ontogeny of the heart describes its development from the fetal to the adult stage. In newborn mammals, blood pressure and thus cardiac performance are relatively low. The cardiomyocytes are thin, and with a central core of mitochondria surrounded by a ring of myofilaments, while the sarcoplasmic reticulum (SR) is sparse. During development, as blood pressure and performance increase, the cardiomyocytes become more packed with structures involved in excitation–contraction (e-c) coupling (SR and myofilaments) and the generation of ATP (mitochondria) to fuel the contraction. In parallel, the e-c coupling relies increasingly on calcium fluxes through the SR, while metabolism relies increasingly on fatty acid oxidation. The development of transverse tubules and SR brings channels and transporters interacting via calcium closer to each other and is crucial for e-c coupling. However, for energy transfer, it may seem counterintuitive that the increased structural density restricts the overall ATP/ADP diffusion. In this review, we discuss how this is because of the organization of all these structures forming modules. Although the overall diffusion across modules is more restricted, the energy transfer within modules is fast. A few studies suggest that in failing hearts this modular design is disrupted, and this may compromise intracellular energy transfer.

This article is part of the theme issue ‘The cardiomyocyte: new revelations on the interplay between architecture and function in growth, health, and disease’.

## 1. Introduction

The heart must work continuously to pump blood around in the body to reach all the capillaries, where it provides the tissues with oxygen and nutrients as well as takes away CO_2_ and waste products. The pumping action is performed by cardiomyocytes, specialized cells with myofibrils that contract to perform mechanical work in response to an elevation of Ca^2+^ during the excitation–contraction (e-c) coupling. As the heart must work continuously, contracting to pump blood and relaxing to refill, for energy supply, it depends mainly on ATP from oxidative phosphorylation in the mitochondria.

In newborn mammals, cardiac performance is relatively low. As it increases during ontogeny, the cardiomyocytes change morphology, e-c coupling and energetics to enhance performance. As there are relatively few studies on neonatal mammals, we can learn a lot from studying cardiomyocytes from other species with similar characteristics. In particular, the cardiomyocytes from fishes bear resemblance to cardiomyocytes from neonatal mammals. In the following, we will describe the ontogeny of cardiomyocytes in mammals in terms of morphology, e-c coupling and energy transfer. When possible, we will draw parallels between cardiomyocytes from neonatal mammals and fishes.

In most mammal species, cardiac performance in terms of stroke work is lower at birth than in adults. During development, blood pressure and cardiac output increase, and thus the heart must perform more mechanical work. In rats, resting diastolic blood pressure increases from approximately 20 to approximately 60 mmHg [1]. In rabbits, mean arterial blood pressure doubles from 50 mmHg at two weeks of age to 100 mmHg in adults [2]. In mice, the mean arterial blood pressure is 30 mmHg at birth, and increases to 80 mmHg in the adult [3,4]. Overall, during development the mammalian heart enhances its performance to generate a three times higher blood pressure. In order to produce these higher pressures and fuel the greater mechanical work, cardiomyocytes change their morphology.

The pressure developed by the left ventricle in neonatal mammals is similar to the mean ventral aortic blood pressure of 30–50 mmHg in trout, cod and several other fish species [5–7]. This is interesting to note, when we throughout the text (see below) compare cardiomyocytes from neonatal mammals and fishes. Although fishes are very different from mammals and operate at lower and varying body temperatures, it is interesting that the cardiomyocyte morphology, e-c coupling and energetics are similar to that of neonatal mammals, which produce similar arterial pressures. This is, of course, a simplification, as the hearts of some fish species produce much lower pressures [7], whereas the heart of the very active tuna can produce mean aortic pressures up to 90 mmHg [8]. The latter is close to adult mammalian levels, while the tuna cardiomyocytes retain the overall morphology of fish cardiomyocytes [9]. It is likely that wall stress of the ventricle has a greater influence on morphology than the absolute pressure. In fish heart, the inner, spongy myocardium divides the ventricle into several smaller luminae with smaller wall stress than the large, central lumen seen in mammalian hearts [10,11]. For simplicity, we mainly compare neonatal mammalian cardiomyocytes with cardiomyocytes from trout, as this is one of the most studied fish species and the one we have used for studies of energy transfer.

## 2. Changes in morphology during ontogeny

**Figure 1.**
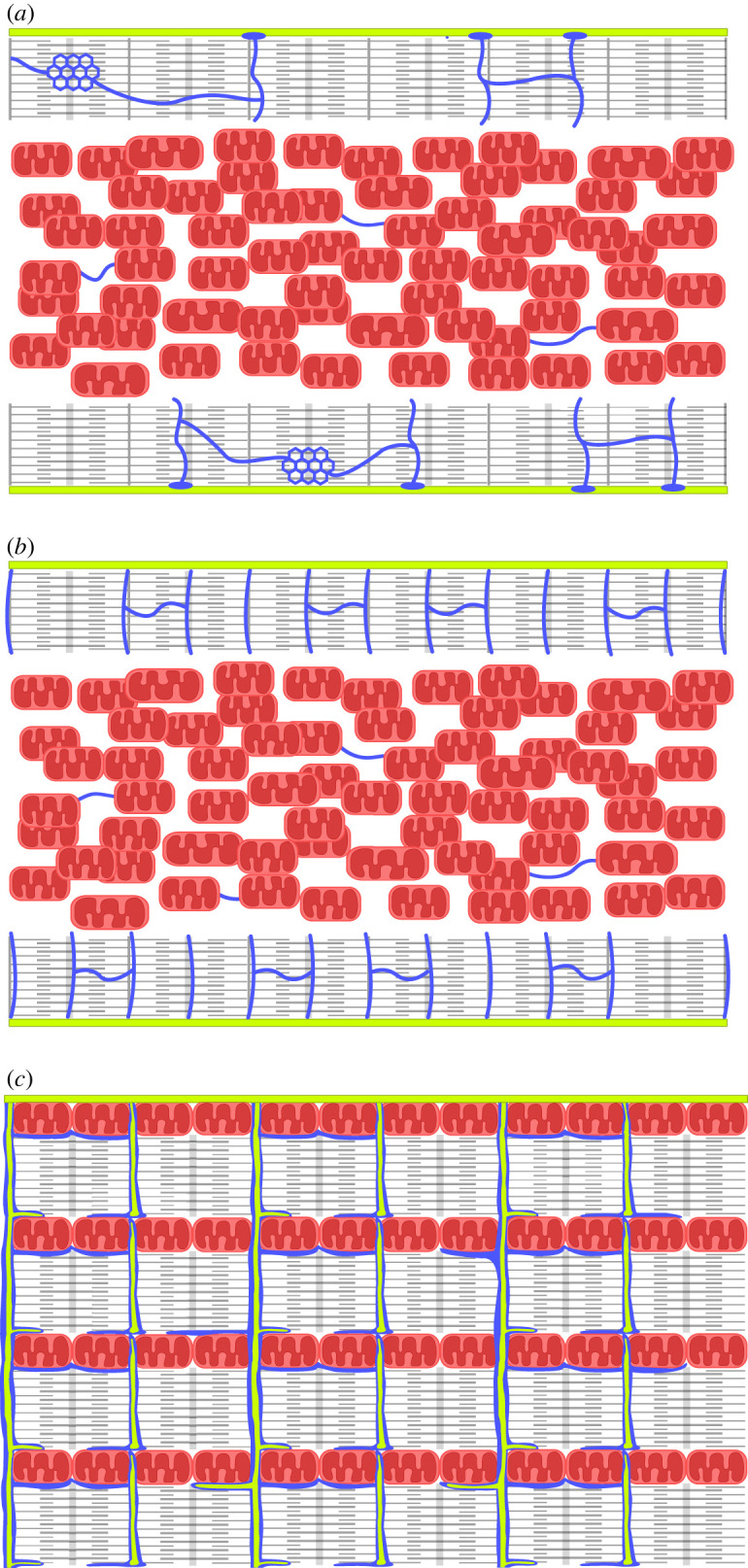
Schematic of (*a*) fish cardiomyocyte, (*b*) neonatal mammalian cardiomyocyte, and (*c*) adult mammalian cardiomyocyte drawn on the basis of the references in table 1. The sarcolemma is shown in green, the myofilaments in grey, the SR in blue, and mitochondria in red. Neonatal mammalian and fish cardiomyocytes have a smooth sarcolemma, which is shown on the top and bottom, because their diameter is relatively small. The myofilaments are situated peripherally, as a ring surrounding the central core of mitochondria. The SR is more irregular in fish cardiomyocytes, where it has no specific relation to the myofibrillar bands, whereas in neonatal cardiomyocytes, the SR is peripheral with the periodicity corresponding to the z-lines and m-band. Adult mammalian cardiomyocytes are thicker and with more internal membrane structures. The sarcolemma invaginates to form transverse tubules (t-tubules). It is only shown on the top side of the figure, because the cell is four to five times wider than fish and neonatal cardiomyocytes. There are multiple, parallel, interchanging rows of myofilaments and mitochondria. The SR wraps around t-tubules, myofilaments and mitochondria.

Figure 1 illustrates the differences between neonatal mammalian, fish, and adult mammalian cardiomyocytes. Their overall characteristics are listed in table 1. Cardiomyocytes from neonatal mammals are spindle-shaped and with a smooth cell membrane, which in muscle cells is called the sarcolemma. They have a diameter of 5–10 µm [26,39,40]. Inside the cell is a central core of mitochondria surrounded by a ring of myofilaments. Mitochondria and myofilaments each take up approximately 30% of the cell volume [30]. The sarcoplasmic reticulum (SR) is present, but sparse and looks to be positioned mainly just below the sarcolemma [26]. This resembles the overall morphology of cardiomyocytes from most fishes, including trout, mackerel and tuna [9,10,15,21,25,36,38].

**Table 1.** Characteristics of fish, neonatal and adult mammalian cardiomyocytes. (The characteristics in this table were used for the schematic drawings in figure 1 to illustrate the similarities between fish and neonatal mammalian cardiomyocytes. The values for fish cardiomyocytes are mainly from papers on trout. However, for the quantitative assessment of SR area per cell volume, data was only found from tuna, where the SR is expected to be more extensive than in most other fish species. The values for adult mammalian cardiomyocytes are mainly from rat and mouse. Within each table cell, references are in square brackets.)

	fishes	neonatal mammals	adult mammals
ventricular morphology	outer compact and inner spongy layer, trabecular sheets form smaller luminae radiating from the central lumen [10–12]	compact wall and central chamber [13]	compact wall, central chamber
mean aortic pressure (mmHg)	40–60 (trout) [5]	approximately 30 [3,4]	approximately 100 [14]
cardiomyocyte length (µm)	100–170 [15–17]	70 [18]	120–140 [18–20]
cardiomyocyte diameter (µm)	4–8 [15–17,21,22]	8 [18]	22–32 [18–20]
cardiomyocyte volume (pl)	1.1–3.4 [15–17]		30–35 [20]
t-tubules	No	No	Yes
sarcolemma µm^2^ area µm^−^^3^ cell vol	approximately 1.2 [15,16,23]	1.05 [24]	4.5–8.5 [20]
myofibril positioning	usually a single peripheral ring [10,21,22,25]	single peripheral ring [26,27]	multiple rows throughout the cell [28]
myofibrillar volume	45–55% [10,29]	30% [30]	55–60% [30]
SR positioning	mainly peripheral, but some in the cytoplasm [9,10,21]	mainly peripheral [26]	continuous network throughout the cell, junctional SR associated with the t-tubules is connected by network SR [28,31]
SR µm^2^ area µm^−^^3^ vol	0.15–0.25 (tuna) [9,32]	0.18 [24]	0.27–1 [19,24,33]
SR contribution to Ca^2+^ fluxes in e-c coupling	highly variable between species, 50% in trout [34]	40% [35]	70–90% [19,35]
mitochondrial positioning	central core, sometimes a few peripheral [9,10,16,21,25,36]	central core [26,27]	subsarcolemmal, perinuclear and intermyofibrillar [36,37]
mitochondrial volume	22–45% [9,15,16,23,29]	32% [30]	31–40% [19,30]
apparent K_M ADP_ of respiration	100–200 [25,38]	70–90 [18,30]	250–300 [18,30]

**Figure 2.**
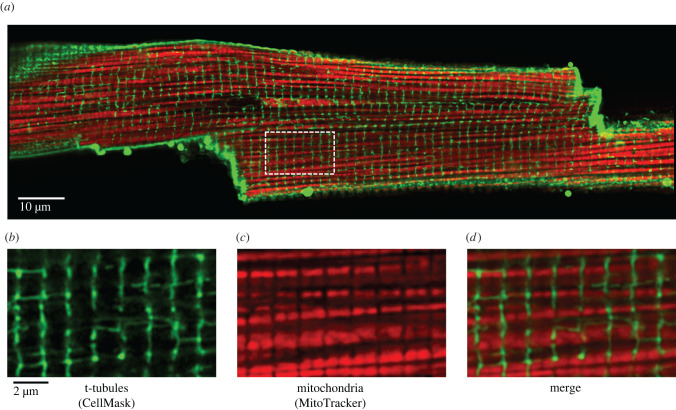
Organization of mitochondria and t-tubules in adult rat cardiomyocytes. (*a*) Overall confocal image of a live cardiomyocyte. Mitochondria (red) were labelled for 10 min with 250 nM MitoTracker® Deep Red FM (ThermoFisher). The sarcolemma (green), including t-tubules, was labelled with 500 nM CellMask™ Orange (ThermoFisher). (*b–d*) Zoom of the white rectangle in panel (*a*), showing the t-tubules (*b*), the mitochondria (*c*) and the merged image (*d*). Note the regular pattern of mitochondria, the highly organized network of t-tubules, and how densely these structures are packed.

During development, the mammalian cardiomyocytes grow in size and change shape to become cylindrical. The main cylinder of the cells has a diameter of 20–30 µm. They sometimes branch and can thus connect to several adjacent cells through the intercalated discs at the end of each branch. As the cells grow in size, the sarcolemma forms invaginations termed transverse tubules (t-tubules) [26,39,41]. The t-tubules branch within the cell to form an elaborate system of both transversal and longitudinal tubules, which are continuous with the sarcolemma, but have a higher density of proteins involved in e-c coupling [42,43]. Owing to the t-tubules, the sarcolemmal surface area relative to the cell volume is much larger in adult mammalian cardiomyocytes (table 1). In mature cardiomyocytes, we find the characteristic crystal-like pattern of the mitochondria, as they are organized in a regular pattern in between the multiple rows of myofilaments [36,37]. For each sarcomere, there is sometimes one large, but more often two small mitochondria delimited by the t-tubules running along the z-lines. This is shown in figure 2, which is a confocal image of a live cell in which the mitochondria and the sarcolemma are labelled in red and green, respectively. The alternating rows of mitochondria and myofilaments take up 30–40% and 50–60%, respectively, of the cell volume [30,44]. The SR takes up a relatively small volume, but it forms an extensive, continuous membrane network throughout the cell [28] and has a relatively large surface area [44].

## 3. Changes in excitation-contraction coupling during ontogeny

The e-c coupling changes in conjunction with the morphology of the cardiomyocytes. As the heart rate increases, the action potential (AP) shortens [41]. In adult cardiomyocytes, the AP triggers Ca^2+^ influx into the cytosol across the sarcolemma through L-type Ca^2+^ channels (LTCCs). This Ca^2+^ influx triggers the Ca^2+^ induced Ca^2+^ release (CICR) from the SR through ryanodine receptors (RYRs). For the cell to relax again, Ca^2+^ is removed from the cytosol by the sarcoendoplasmic reticulum Ca^2+^ ATPase (SERCA), the Na^+^/Ca^2+^-exchanger (NCX) operating in forward mode and the plasma membrane Ca^2+^ ATPase (PMCA). During development, there is a change in the relative contribution of these Ca^2+^ flux pathways.

In neonatal cardiomyocytes, Ca^2+^ enters the cell through LTCCs as well as reverse action of the NCX (NCX_rev_). However, NCX_rev_ is the dominant transsarcolemmal Ca^2+^ influx pathway, and the main influx pathway coupled to CICR [35]. This scenario is in line with structural studies showing that in neonatal cardiomyocytes, a larger fraction of NCX and a smaller fraction of LTCC colocalize with RYR, and almost exclusively at the periphery of the cell [26,40]. CICR only contributes to approximately 40% of the Ca^2+^ transient [35]. Owing to the sparse SR and its smaller role in e-c coupling, the Ca^2+^ transients rise faster at the edges of the cell than in the centre [41].

A similar pattern of e-c coupling is found in fishes, where sarcolemmal Ca^2+^-fluxes are also relatively more important than SR Ca^2+^-cycling [45]. The Ca^2+^-fluxes in cardiomyocytes from neonatal rabbits and fish are similar. In trout cardiomyocytes, NCX_rev_ is also the main transsarcolemmal Ca^2+^ influx pathway contributing approximately 30% of the total Ca^2+^ influx, whereas LTCC contributes approximately 20%, and CICR 50% to the overall Ca^2+^ transient [34]. In trout cardiomyocytes, the Ca^2+^-transient also rises faster at the edges than in the centre of the cell [22]. However, there is great variation between different species of ectothermic vertebrates, which have different behaviour and preferred temperature. In general, active species rely more on Ca^2+^-cycling through the SR than sedentary species [45].

In adult mammalian cardiomyocytes, the e-c coupling changes to become more reliant on CICR from the SR triggered by Ca^2+^-influx through LTCCs. Concomitant with the development of t-tubules, the co-localization of NCX and RYR gradually declines [26], while co-localization of LTCC and RYR increases, and a larger fraction of the LTCC-RYR couplings are internal, along the t-tubules, rather than peripheral, on the cell surface [39,40]. CICR contributes to approximately 70–90% of the Ca^2+^ transient [19,35], and the Ca^2+^ transients are spatially homogeneous, with a synchronous rise at the edges and in the centre of the cell [41].

The changes in e-c coupling, that are observed during ontogeny, rely not only on changes in protein expression, but to a large extent also on the cardiomyocyte morphology. As noted above, the SR forms a continuous network throughout adult mammalian cardiomyocytes [28], and the close contacts of the SR network with the sarcolemma are crucial for adequate Ca^2+^ dynamics. In adult cardiomyocytes, the majority of LTCCs in the sarcolemma and RYRs in the junctional SR co-localize internally, at the t-tubules [40], where the close association of the sarcolemma and SR membranes leads to the formation of the dyadic space. Within the dyadic space, LTCCs and RYRs form couplons, which are only approximately 12 nm apart [46,47]. Within these couplons are Ca^2+^ microdomains in which the Ca^2+^ concentrations are much higher than in the surroundings, and this is critical for the LTCC regulation of CICR. Indeed, Ca^2+^ release events are delayed in loose dyads, in which the distance between RYR and LTCC is larger than in compact dyads [48]. An increased number of orphaned RYR clusters outside of dyadic space leads to dyssynchrony of the Ca^2+^ transient [49–51]. Thus, the formation of t-tubules regulated by BIN1 (amphiphysin) and the tight coupling between LTCC and RYR held in place by junctophilin is the structural prerequisite for a rapid, synchronous Ca^2+^ release and, thus, contraction [52–54].

The Ca^2+^-fluxes in e-c coupling change with adrenergic stimulation enhancing cardiac performance during the fight-or-flight response. Stimulation of β-adrenergic receptors interacting with G_s_-protein activates adenylyl cyclase to produce cyclic AMP (cAMP), which in turn binds to and activates protein kinase A (PKA). PKA phosphorylates several proteins involved in e-c coupling such as the LTCC, RYR, phospholamban and troponin I. This results in an increased LTCC open probability and Ca^2+^-influx [55] and a faster SR Ca^2+^ uptake, as the phosphorylation of phospholamban removes its inhibition of SERCA [56]. The result is a positive inotropic, lusitropic and chronotropic effect, i.e. increased contraction, enhanced relaxation and faster heart rate, respectively. This functional adrenergic response has been well studied in adult mammalian cardiomyocytes, but few studies have addressed this in neonatal mammalian cardiomyocytes, and we have not been able to find a direct comparison of whether adrenergic stimulation affects neonatal and adult mammalian cardiomyocytes differently. In neonatal cardiomyocytes, adrenergic stimulation increases the LTCC Ca^2+^-current [57], but although phospholamban is phosphorylated, this does not always affect the Ca^2+^ transients, reflecting the functional state of the SR [58]. Adrenergic stimulation also increases the LTCC Ca^2+^-current in fish cardiomyocytes, but the magnitude of the response is species-dependent [15,59]. In fishes with a relatively well developed SR, adrenergic stimulation also leads to greater recruitment of the SR, but it is uncertain whether this is owing to the larger LTCC Ca^2+^-influx [60], a direct effect on RYR and phospholamban, or both.

## 4. Changes in metabolism during ontogeny

Whereas energy for the ion fluxes during excitation comes from their electrochemical gradient, the re-establishment of ion gradients during relaxation costs energy in the form of ATP. SERCA consumes 1 ATP per 2 Ca^2+^ and PMCA consumes 1 ATP per Ca^2+^. The NCX does not consume ATP directly but is coupled through the electrochemical gradient of Na^+^ to the Na^+^/K^+^ ATPase, and thus indirectly consumes 1 ATP per Ca^2+^ transported out of the cell. Usually, the main consumption of ATP is by myosin ATPase as the sarcomeres contract. However, this varies with the mechanical load, heart rate and inotropic state. The ATP consumption of actomyosin is directly related to the pressure developed by the heart and the volume of blood pumped, and there is a linear relationship between cardiac oxygen consumption and the pressure–volume area of the working heart [61]. It has been estimated that of the ATP consumed by the heart, approximately 60% was for myosin ATPase [61,62], 30% for e-c coupling, and approximately 10% for basal metabolism [63].

The sources of ATP in the heart also change during ontogeny. In the fetal stage, cardiac metabolism is adapted to the slightly hypoxic conditions *in utero*, and hearts of newborn mammals are more hypoxia-tolerant than in the adult stage [64]. Of the total ATP, approximately 40% is generated by glycolysis, approximately 45% is generated by oxidative phosphorylation of glucose and lactic acid, while the remaining approximately 15% is generated by oxidative phosphorylation of fatty acids [65]. Fish also have a higher glycolytic capacity and are more hypoxia-tolerant than mammals [66,67]. After birth, mammalian cardiomyocytes exhibit a shift in metabolism. In most species, cardiac oxygen consumption increases during development, and the cardiomyocytes rely increasingly on the uptake and oxidation of fatty acids [65]. In the adult mammalian heart, 70–95% of ATP is generated by oxidative phosphorylation in the mitochondria [62].

## 5. Energy transfer and the creatine kinase system

A key question in cardiac energetics concerns energy transfer, i.e. how ATP and ADP circulate between ATPases and mitochondria, and whether the energy transfer changes in response to the changes in intracellular structure during the development. Energy transfer can occur by direct diffusion of ATP and ADP, and/or by creatine kinase (CK) facilitated diffusion.

CK catalyses the following, reversible reaction: ADP + phosphocreatine + H^+^ ↔ ATP + creatine. In the adult mammalian heart, there is one mitochondrial isoform (Mi-CK) and three cytosolic isoforms (MM, MB and BB-CK). Mi-CK is bound to the outer surface of the inner mitochondrial membrane and forms complexes with the voltage-dependent anion channel (VDAC) in the outer mitochondrial membrane (OMM) and adenine nucleotide translocase (ANT) in the inner mitochondrial membrane [68]. The cytosolic MM-CK is structurally bound near the main ATPases in cardiomyocytes, such as myosin ATPase [69], SERCA [70], the Na^+^/K^+^ ATPase [71] and the ATP-sensitive potassium channel, K_ATP_ [72]. The binding of MM-CK near these ATPases in the cardiomyocytes makes it an efficient buffer of the phosphorylation potential, which determines the amount of energy released by ATP hydrolysis [68]. Indeed, the CK system is recognized as a temporal energy buffer, using phosphocreatine to regenerate ATP at times when ATP consumption exceeds energy generation.

The role of CK in the heart is still a subject of research. It was suggested to be more important as a spatial energy buffer facilitating energy transfer between mitochondria and ATPases, because it generates an additional diffusional circuit between ATPases and mitochondria [73]. Furthermore, creatine and phosphocreatine diffuse faster than ADP and ATP, and are present in relatively high concentrations, which allows build-up of larger diffusion gradients [68] without impacting the phosphorylation potential. This could be important in cells with long diffusion distances between ATPases and mitochondria, or with intracellular diffusion barriers restricting diffusion [74].

## 6. Use of the apparent K_M ADP_ of mitochondrial respiration to probe intracellular diffusion

As probably the simplest way to assess diffusion in cardiomyocytes, the apparent K_M ADP_ is estimated from recordings in the respirometer, where the respiration of permeabilized fibres or cardiomyocytes is recorded while stepwise increasing the concentration of ADP until the respiration rate is maximal. Permeabilized fibres are prepared from small pieces of the heart wall, which are gently dissected with fine tweezers to separate small bundles of cells [75]. Fibres or isolated cardiomyocytes are then treated with saponin, which selectively permeabilizes the sarcolemma allowing the experimenter to affect the intracellular environment [76]. If the permeabilized fibres or cardiomyocytes are provided with substrates for the citric acid cycle (typically glutamate, malate, pyruvate and succinate), the respiration rate increases upon addition of ADP. The apparent K_M ADP_ is the ADP concentration that stimulates respiration to half of the maximum respiration rate. In isolated mitochondria, which are taken out of their structural setting, the apparent K_M ADP_ is 5–20 µM, but permeabilized fibres or cardiomyocytes from adult mammals have a higher apparent K_M ADP_ of approximately 200–400 µM [18,73,75,77–79]. This difference between isolated mitochondria and permeabilized fibres or cells is frequently attributed to the diffusion restrictions imposed by intracellular structures. Indeed, when recorded in the respirometer, the apparent K_M ADP_ indicates the difficulty with which ADP diffuses from the medium outside the cells to the mitochondrial inner membrane [80], and the measurements correspond to the integrated response of all mitochondria in the cell, including mitochondria from the most central region to the parts of the cell close to the sarcolemma.

The higher apparent K_M ADP_ in permeabilized cells compared to isolated mitochondria indicates that this overall diffusion is restricted [75], as the K_M ADP_ would correspond to the concentration gradient between the solution and the mitochondrial inner membrane required to sustain the mitochondrial ADP-consumption through ADP diffusion. From outside the cells, ADP can encounter several barriers that may prolong or hinder the diffusion. It was suggested that unstirred layers surrounding permeabilized fibres result in unphysiologically long diffusion distances before ADP in the solution has even encountered the cells [81]. In addition, permeabilized fibres can form aggregates leading to longer diffusion distances than assumed for separate cells. However, by combining experimental and modelling approaches, it was demonstrated on single permeabilized cardiomyocytes from rat heart that also when taking unstirred layers into account, the high apparent K_M ADP_ is caused by barriers inside the cells [82]. Difference in diffusion distance, though, was found when comparing the apparent K_M ADP_ in permeabilized trout fibres and cardiomyocytes with fibres having a much higher K_M ADP_ [38]. This was attributed to the difficulties in preparation of the fibres from such thin and fragile cells as trout cardiomyocytes leading to relatively thick fibres [38]. In addition to diffusion distances, the apparent K_M ADP_ can be impacted by the relative activities of mitochondrial ATP synthesis and surrounding ATPases. As shown by Kongas *et al.* [83], increasing the endogenous ATPase activity can lead to a reduction of the apparent K_M ADP_.

Thus, while estimating the apparent K_M ADP_ is very useful to get an impression of the overall diffusion restrictions within the cell, experiments on fibres or where the relative contributions of ATP synthesis and ATP consumption could change, such as during a treatment, must be interpreted with caution. However, as noted above, quantitative analyses of data from isolated, permeabilized rat cardiomyocytes have demonstrated the intracellular nature of the diffusion barriers. Knowing the identity and location of these barriers is important for understanding the physiological role of the CK system.

## 7. Creatine kinase and the apparent K_M ADP_

In the 1990s, it was suggested that the high apparent K_M ADP_ in adult cardiomyocytes was owing to diffusion restriction caused by the OMM. The physiological advantage would be that energy transfer of ADP/ATP between mitochondria and ATPases would take place through the CK system [84]. Diffusion restriction by the OMM leads to the formation of an isolated compartment in the mitochondrial intermembrane space with concentrations of ADP/ATP differing from those outside the mitochondria. This would enhance the generation of phosphocreatine by Mi-CK [85] in addition to the direct transfer of ATP and ADP between ANT and Mi-CK [86]. Thus, it was suggested that a relatively impermeable OMM would let most of the energy transfer take place through the CK system, and this was, as noted above, beneficial in terms of faster diffusion and rapid buffering of the phosphorylation potential near ATPases.

The importance of CK as a facilitator of energy transfer in muscles with intracellular diffusion barriers was supported by the finding that its acute inhibition lowered the phosphorylation potential near ATPases to such an extent that cardiac function was impaired [87]. Furthermore, a comparison of different muscle types found that with an increase in aerobic capacity, there is a concomitant increase in the apparent K_M ADP_ of respiration as well as Mi-CK expression [73,88]. Thus, when comparing different types of muscles, the expression of Mi-CK correlates with the increase in apparent K_M ADP_. Again, this backed up the theory that diffusion restriction by the OMM shifted energy transfer to take place through the CK system.

## 8. Energy transfer when creatine kinase is inhibited: lessons from transgenic mice

There are several transgenic mouse models, in which CK is inhibited by knockout of its different isoforms (CK KO [89]), or of the enzymes involved in creatine synthesis and uptake (arginine : glycine amidinotransferase [90], guanidinoacetate methytransferase (GAMT, [91]) and creatine transporter [92], respectively). Inhibition of CK affects the skeletal muscle phenotype [89,93–96], but studies on the heart have been equivocal [97] and should be interpreted with caution [98]. In general, cardiac performance of CK KO and GAMT KO mice is normal at baseline levels and only compromised at very high workloads [99–102].

If diffusion restriction by OMM caused channelling of energy transfer through the CK system, and this was crucial for cardiomyocyte function, it is conceivable that inhibition of the CK system would lead to compensatory changes in the permeability of the OMM. In permeabilized cardiac fibres from transgenic mice lacking Mi-CK, the apparent K_M ADP_ was the same as in wild-type (WT) [103]. In fibres from CK KO mice lacking both the cytosolic and mitochondrial CK isoforms, the apparent K_M ADP_ was lower than in WT, and mitochondria seemed to intercalate between the myofibrils [104]. However, in cardiomyocytes from creatine-deficient GAMT KO mice, where the CK system is inhibited by lack of creatine, the apparent K_M ADP_ as well as mitochondrial organization was the same as in WT littermates [105], and there were no changes in alternative energy transfer systems [106]. Thus, in transgenic mouse models with an inhibited CK system, there is no clear correlation between the function of Mi-CK and the apparent K_M ADP_. This is because the OMM is not the only diffusion barrier within cardiomyocytes.

## 9. Intracellular diffusion barriers form modules within the cells

More recently, it has become clear that intracellular diffusion restriction is not just owing to the OMM but has multiple causes. The search for multiple factors imposing diffusion restrictions intensified in 2001, when experimental works demonstrating that there is an unexpected coupling between ATPases and mitochondria in the cardiomyocytes were published [104,107,108] leading to the declaration that the cytoplasm in the heart cannot be considered as a well-mixed bag anymore [109]. Diffusion barriers are formed by membrane structures as well as protein dense areas. The main barriers are the OMM and the SR [110]. When considering just one mitochondrion and the ATPases around, it may seem counterintuitive that the OMM and SR separate ATPases from the mitochondria and thus hinder energy transfer. However, the barriers are not only at the level of the OMM, but also in the cytosol [86,111]. As noted in the beginning, adult mammalian cardiomyocytes have multiple interchanging rows of myofibrils and mitochondria and a continuous SR connecting to the t-tubules throughout the cell. This overall organization of intracellular membrane structures, as they envelop the myofibrils, leads to the formation of modules. These modules have also been termed intracellular energetic units [108]. The diffusion of ATP and ADP out of these modules is restricted, and ATP/ADP are expected to preferentially circulate only between neighbouring ATPases and mitochondria within the module.

**Figure 3.**
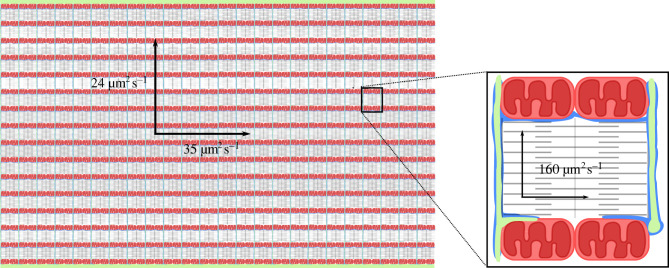
Schematic illustration of a section of an adult mammalian cardiomyocyte. On the left, the cell is shown in full width, but not full length, with sarcolemma (green) on the top and bottom invaginating to form t-tubules. The many parallel rows of mitochondria (red) and myofilaments (grey) in contact with t-tubules as well as the SR (blue) form modules within the cell. Whereas the diffusion coefficient in solution is 200 µm^2^ s^−1^, the overall diffusion coefficient across several modules is 80–90% lower (24 and 35 µm^2^ s^−1^ in the transversal and longitudinal direction, respectively). On the right is shown an enlargement of the black square illustrating a single module, i.e. a sarcomere surrounded by mitochondria, t-tubules and SR. Within each module, the diffusion coefficient is estimated to be 80% of the coefficient in solution, i.e. 160 µm^2^ s^−1^. Diffusion coefficients are from Illaste *et al*. [112].

The modules are not completely isolated from each other, but the barriers between them cause a considerable slowing of the overall diffusion in adult cardiomyocytes. Note, that the term ‘overall diffusion’, means diffusion across several modules. This is shown in figure 3, left panel. The diffusion is anisotropic, being faster in the longitudinal than in the radial direction [113]. An analysis of raster image correlation spectroscopy data from rat cardiomyocytes suggests that diffusion barriers within the cells form a lattice with the dimensions of approximately 0.8 µm in the radial and approximately 0.9 µm in the longitudinal direction [112]. These dimensions are in agreement with the structure of the sarcomeres surrounded by SR and mitochondria with barriers also formed by the t-tubules, m-bands and z-lines [114]. Although diffusion between modules is slow, diffusion within modules is relatively fast, as illustrated in figure 3, right panel. The diffusion coefficient of fluorescently labelled ATP is approximately 300 µm^2^ s^−1^ in water and approximately 200 µm^2^ s^−1^ in physiological solution containing bovine serum albumin. The overall diffusion coefficient is 24 and 35 µm^2^ s^−1^ in the radial and longitudinal direction [112]. This estimate is significantly smaller than ionic mobility found for frog skeletal muscle [115], but very close to the estimations of overall diffusion coefficient through analysis of heterogeneity of mitochondrial autofluorescence (23–30 µm^2^ s^−1^) [111] and imposed heterogeneity of cAMP (32 µm^2^ s^−1^) [116]. While the overall diffusion is relatively slow, the diffusion coefficient within each module is approximately 80% of that in physiological solution, i.e. 160 µm^2^ s^−1^ [112]. Thus, diffusion within each module is approximately five times faster than the overall diffusion measured across multiple modules.

The modular design has the effect that when recording the respiration of permeabilized cardiomyocytes while adding ADP to the solution outside the cells, peripheral modules restrict the diffusion to more central modules. Indeed, when changing the ADP concentration outside the cardiomyocytes, the peripheral mitochondria respond before the central mitochondria, as is observed in pictures of how mitochondrial autofluorescence changes with the concentration of ADP outside the cell [111]. A three-dimensional modelling study of the cardiomyocyte with modules separated by barriers was able to reproduce several data from respiration experiments, including the high apparent K_M ADP_ [117]. This also explains why the mitochondria have a higher affinity for endogenous ADP generated by ATPases than for exogenous ADP added to the solution outside the cells [107]. Thus, the apparent ADP-affinity recorded in permeabilized cardiomyocytes is only apparent, and it cannot be extrapolated to represent the ADP-affinity of individual mitochondria within the cell.

There are several advantages of the modular design of cardiomyocytes. In terms of energy transfer, it reduces diffusion distances and leads to what is termed ‘direct adenine nucleotide channelling’. In adult cardiomyocytes, direct adenine nucleotide channelling from mitochondria to myosin ATPase and SERCA is almost or as efficient a source of ATP as the CK system [30,104]. In addition, the modular design can have a protective effect when things go wrong. For example, within a cardiomyocyte, individual mitochondria may experience oscillations of the membrane potential without affecting the neighbouring mitochondria [118]. Although healthy mitochondria are reported to be connected in a large reticulum [119], their network is dynamic, and malfunctioning mitochondria are physically separated from their neighbours [120]. This allows damaged mitochondria to be removed by autophagy and replaced with new and fully functional mitochondria [121]. While this may affect the contractile performance of the sarcomere within the module in question, the diffusion barriers between modules protect neighbouring modules. Thus, if there is minor damage within individual modules, they can be replaced without too much effect on the rest of the cell. This is particularly important in cardiomyocytes, which are terminally differentiated. As they cannot divide and proliferate, damaged cells cannot be replaced, but modules within the cells can be renewed.

While most of the modelling approaches focus on intermyofibrillar mitochondria for the sake of simplicity, it should be noted that during the functional recordings of respiration or autofluorescence, it is not possible to distinguish them from perinuclear or subsarcolemmal mitochondria. The three different subpopulations of mitochondria have their own morphology and biochemistry [122]. As subsarcolemmal and a large part of the perinuclear mitochondria are also positioned next to sarcomeres in addition to other local ATPases, we expect that all three subpopulations of mitochondria participate in the formation of modules. However, the local energy transfer for perinuclear and subsarcolemmal mitochondria warrants further studies.

## 10. Energy transfer within modules

The barriers between modules explain approximately 50% of the overall diffusion restriction with the remaining 50% being at the level of the OMM, but it is not possible to distinguish whether it is owing to the SR and/or the OMM [111]. The OMM is permeable through the pores formed by the VDAC. Indeed, VDAC has been termed the ‘gatekeeper’ of the OMM [123], and its voltage-sensitivity and permeability is regulated by multiple factors such as tubulin-binding [124], hexokinase interaction [125], and glutamate [126,127]. The heart expresses three different isoforms of VDAC (VDAC1, 2 and 3) with VDAC1 and VDAC2 being the dominant isoforms [128]. The role of the VDAC is not only to regulate the access of ATP and ADP to the mitochondria. It also regulates the access of many other molecules such as ions, NADH and substrates for the citric acid cycle (pyruvate from glycolysis and fatty acids). In particular, the importance of VDAC in the regulation of mitochondrial Ca^2+^-uptake is receiving increased attention [129,130].

The structural prerequisite for mitochondrial Ca^2+^-uptake is a close association between the SR and the mitochondria. This allows the formation of microdomains with high Ca^2+^ concentrations, as is necessary for Ca^2+^ uptake through the mitochondrial Ca^2+^ uniporter, which has a low Ca^2+^ affinity of 10–30 µM [131]. To the best of our knowledge, the SR-mitochondria microdomain Ca^2+^ concentration has not been measured in cardiomyocytes, but mathematical modelling suggests that mitochondrial Ca^2+^-uptake does not play a role in e-c coupling on a beat-to-beat basis [132]. However, it stimulates several dehydrogenases in the citric acid cycle as well as the ATP synthase and may be important for the regulation of ATP generation [133]. Three-dimensional high-resolution images of intracellular structures in cardiomyocytes suggest that the SR and mitochondria are juxtaposed mainly near the t-tubules, where transversal branches of SR wrap the t-tubules on one side and connect to the mitochondria on the other side. Some juxtaposition is also observed where longitudinal branches of the SR follow the perimeter of the mitochondria, connecting neighbouring transversal branches [28,134]. In the places where the SR is juxtaposed to the mitochondria, the RYRs and mitochondria are up to approximately 200 nm apart [135]. On the one hand, the SR may shield the mitochondria from ADP from the myofilaments, but on the other hand, it allows for the direct energy transfer between mitochondria and SERCA that has been observed in cardiomyocytes [104]. Furthermore, the SR may be associated with the mitochondria for other purposes than Ca^2+^ transport and energy transfer. Some studies have suggested that the SR is continuous with the endoplasmic reticulum (ER), and may carry out the same functions such as protein synthesis and folding [136]. This is in agreement with a recent paper demonstrating ribosomes localized near the z-lines throughout adult rat cardiomyocytes [137]. Thus, the SR-mitochondria contacts in cardiomyocytes may serve the same purpose as mitochondria-associated ER membranes in other cell types, where they participate in, for example, lipid synthesis and transfer, mitochondrial dynamics, and autophagy [138]. The extent of shielding depends on how large a fraction of the mitochondrial surface area is shielded by the SR. In HeLa cells, it was estimated that 5–20% of the mitochondrial network surface area is in close contact with the SR [139]. It would be very interesting to see a similar quantitative analysis from cardiomyocytes of how large a fraction of the mitochondrial membrane is closely associated with the SR.

Recent studies suggest that submitochondrial heterogeneity in VDAC isoform distribution should also be considered. Although the roles of the different VDAC isoforms may to some extent be overlapping, they are also distinct. It seems that VDAC1 interacting with IP_3_-receptors, and VDAC2 interacting with RYR2 are aimed at Ca^2+^ shuttling, whereas VDAC1 interaction with ANT and hexokinase is aimed at metabolite shuttling [130]. It is likely that submitochondrial localization of different VDAC isoforms is also involved in the regulation of their function, and it is an appealing hypothesis that in the regions of mitochondria-SR contact, VDAC2and RYR2 form couplons for exchange of Ca^2+^, while outside these contact regions, VDAC1 and ANT form couplons for exchange of ATP/ADP [130]. If most of the VDACs aimed at metabolite shuttling are outside the regions of mitochondria-SR contact, then shielding by the SR would have a relatively small impact on the energy transfer between myofilaments and mitochondria. Experiments demonstrating that mitochondria are almost or as good a source of ATP for myofibrillar contraction as CK [30,104] suggest that shielding by the SR does not hinder energy transfer between mitochondria and myosin ATPase function to a significant extent.

If the diffusion restriction by the OMM does not limit the performance of myosin ATPase and SERCA, it raises questions about the physiological role of the CK system. As noted above, transgenic mouse models suggest that Mi-CK is not needed to facilitate energy transfer across the OMM, at least at baseline levels of activity. This speaks in favour of that the main role of the CK system in the heart is as a temporal energy buffer, regenerating ATP when the ATP-demand exceeds the ATP generation by mitochondria. This could happen during abrupt changes in workload such as during a fight-or-flight response. The role of the CK system as a spatial energy buffer is still not clear. It is conceivable that spatial energy buffering might be necessary at high workloads. As noted above, the performance of CK and creatine-deficient hearts is limited at high workloads [99,102]. Measurements using nuclear magnetic resonance magnetization transfer have shown that, under some conditions, energy transfer between mitochondria and ATPases is carried via combined diffusion of ATP and phosphocreatine. However, owing to the noise limitations, it was impossible to get more specific estimates of energy transfer via CK for different heart workloads [140]. In order to quantitatively estimate CK contribution through mathematical models, we need to know more about the distribution and dynamics of diffusion barriers within cardiomyocytes. Indeed, most, if not all, of the studies on diffusion barriers within the cell were performed on relaxed, quiescent permeabilized fibres or cardiomyocytes with glutamate and malate as respiratory substrates. This hardly reflects the situation *in vivo*, where the cardiomyocytes receive multiple substrates for the citric acid cycle and oxidative phosphorylation. Glutamate reduces the open probability of VDAC [126], and it is conceivable that the substrate dependency of the apparent K_M ADP_ [141] is owing to substrate regulation of VDAC open probability. Furthermore, the cardiomyocytes *in vivo* continuously contract and relax against a load. Tubulin, being an important part of the cytoskeleton, is a known regulator of VDAC permeability [124] and could be a mediator of mechanical regulation of VDAC open probability. Therefore, while recordings on permeabilized cardiomyocytes can give us an idea about the communication within the cells, they cannot be extrapolated to the situation *in vivo*. In permeabilized cardiomyocytes, the fraction of VDAC molecules that were accessible to ADP was surprisingly small, approximately 2% [111], but this is likely to be different and dynamic in the working heart.

## 11. Changes in diffusion barriers and energy transfer during ontogeny

As noted in the beginning, cardiomyocytes from newborn mammals are slender and have a single, peripheral ring of myofilaments surrounding a central core of mitochondria, and a sparsely developed SR. Their morphology is very similar to that of trout cardiomyocytes (figure 1 and table 1). In permeabilized fibres from neonatal mammals, the apparent K_M ADP_ is approximately 80 µM [30]. As measurements on fibres can be difficult to interpret, it is notable that isolated, permeabilized trout cardiomyocytes have an apparent K_M ADP_ of 100–200 µM [25,38]. This suggests that although these cardiomyocytes lack the modular structure, there are still barriers present restricting the diffusion of ADP from the medium to the mitochondrial inner membrane. This is in agreement with 50% of the overall diffusion restriction being at the level of the OMM [111]. Again, it is not possible to determine whether this is owing to the permeability of the OMM and/or shielding by the SR, but as the SR is less developed, it is tempting to speculate that most of this is owing to the OMM.

In terms of facilitated energy transfer by CK, it should be noted that in neonatal mice and rabbits, Mi-CK activity is low, and it is not yet coupled to respiration [30,142]. Trout cardiomyocytes also have very low expression of Mi-CK [25]. Thus, although there seems to be diffusion barriers at the level of the OMM, they do not need Mi-CK to facilitate energy transfer. This could relate to their lower performance.

During development, the cardiomyocytes loose most of their cytosolic space to become densely packed with t-tubules, myofilaments, SR and mitochondria (figure 1). As the density of structures increases, the apparent K_M ADP_ increases [18,30] indicating a decrease in the overall diffusion inside the cells (table 1). However, the structures form modules keeping energy transfer local between adjacent mitochondria and ATPases. Thus, as the cardiomyocytes develop multiple interchanging rows of myofibrils and mitochondria, the positioning of intermyofibrillar mitochondria brings the source of ATP to where it is needed to fuel the contraction of the cardiomyocyte.

## 12. Changes in diffusion barriers in disease

There are few functional studies of what happens to the diffusion barriers in diseased hearts. After acute ischaemia and coronary artery ligation, the apparent K_M ADP_ is lower [143,144] indicating a loss of diffusion barriers. This can be partially explained by rupture of the OMM [143,144], but changes in the overall organization of intracellular membrane structures should also be considered. Although there may be some differences depending on the aetiology, cardiomyocytes from failing hearts, overall, exhibit swelling and loss of t-tubules, clusters of mitochondria and disorganization of the SR [28,39,49,52]. This would disrupt the modular organization of cardiomyocytes and could have a detrimental effect on energy transfer. This would also explain why the cardiac phenotype of creatine-deficient mice, where the modules are intact, is relatively mild, whereas post-ischaemic and failing hearts benefit from overexpression of CK to facilitate energy transfer [145,146].

## 13. Summary

Whereas most of this review has focused on diffusion barriers and how they govern energy transfer within the cell, it started with a description of how the overall morphology, e-c coupling and energetics change during ontogeny in mammals. In addition, parallels were made between cardiomyocytes from fishes and neonatal mammals. The latter information was included to illustrate the bigger picture: cardiomyocytes with similar workloads—such as in fishes and neonatal mammals—are remarkably similar in both morphology, e-c coupling, energetics and energy transfer. Thus, studies of different species can provide information about general principles in cardiac physiology. As mammals develop, cardiac performance increases and the cardiomyocytes adapt to the higher workload. They grow in diameter and become more structurally packed. Multiple interchanging rows of myofilaments and mitochondria take up approximately 90% of the cell volume and are intersected by t-tubules and the SR. The juxtapositioning of the SR with t-tubules and mitochondria forms microcompartments with higher Ca^2+^ concentrations than in the cytosol, and these are crucial for rapid and adequate Ca^2+^ signalling in e-c coupling. The picture is more complicated when looking at energy transfer, where it is counterintuitive that the OMM and perhaps the SR restrict diffusion of ADP and ATP between ATPases and the mitochondria. However, the overall organization of the SR and mitochondria together with protein dense parts of the sarcomeres form modules within the cells. This modular design keeps diffusion distances relatively short. Thus, whereas e-c coupling relies on microcompartments for better communication through Ca^2+^, energy transfer relies on macrocompartments for a tight communication between ATPases and mitochondria. Studies on mice, in which the CK system is inhibited, suggest that the modular design of cardiomyocytes ensures a sufficient energy transfer at baseline workloads. However, in failing hearts with disorganized structures, energy transfer may be compromised as the modules are disrupted.

## Data Availability

This article has no additional data.
